# Pseudo-Random Encryption for Security Data Transmission in Wireless Sensor Networks

**DOI:** 10.3390/s19112452

**Published:** 2019-05-29

**Authors:** Liang Liu, Wen Chen, Tao Li, Yuling Liu

**Affiliations:** 1 College of Cybersecurity, Sichuan University, Chengdu 610065, China; liangzhai118@163.com (L.L.); litao@scu.edu.cn (T.L.); xiaye524@163.com (Y.L.); 2 College of Electronics and Information Engineering, Sichuan University, Chengdu 610065, China

**Keywords:** wireless sensor network, network security, pseudo-random function, distributed detection, likelihood ratio test

## Abstract

The security of wireless sensor networks (WSN) has become a great challenge due to the transmission of sensor data through an open and wireless network with limited resources. In the paper, we discussed a lightweight security scheme to protect the confidentiality of data transmission between sensors and an ally fusion center (AFC) over insecure links. For the typical security problem of WSN’s binary hypothesis testing of a target’s state, sensors were divided into flipping and non-flipping groups according to the outputs of a pseudo-random function which was held by sensors and the AFC. Then in order to prevent an enemy fusion center (EFC) from eavesdropping, the binary outputs from the flipping group were intentionally flipped to hinder the EFC’s data fusion. Accordingly, the AFC performed inverse flipping to recover the flipped data before data fusion. We extended the scheme to a more common scenario with multiple scales of sensor quantification and candidate states. The underlying idea was that the sensor measurements were randomly mapped to other quantification scales using a mapping matrix, which ensured that as long as the EFC was not aware of the matrix, it could not distract any useful information from the captured data, while the AFC could appropriately perform data fusion based on the inverse mapping of the sensor outputs.

## 1. Introduction

Wireless sensor networks (WSNs) are commonly deployed in various practical applications to gather information from a hostile area or placements where human intervention is impossible, and thus can support many new and important areas, such as customer satisfaction survey [1], unmanned aerial vehicles [2], military surveillance [3], and fire detection [4]. However, due to the broadcast nature of the wireless network, sensor data are prone to be intercepted by unauthorized receivers [5,6]. The security of WSN mainly involve decentralized detection whereby the sensors send their measurements to an ally fusion center (AFC) which attempts to detect the state of nature using the data received from all the sensors, meanwhile an enemy fusion center (EFC), also located in a vicinity of the AFC, tries to eavesdrop on the wireless communications between sensors and the AFC [7].

The data confidentiality of WSN has been the subject of many studies using different security strategies [8,9]. To facilitate data encryption using symmetric cryptographic algorithms, the authors in [10,11] proposed novel lightweight anonymous authentication and key agreement (AKA) protocols for WSN, which combines multi-security stages: User registration, sensor node registration, login, authentication, key agreement, and password change. However, the issue of scalability remains a great challenge, since a sensor’s life span is largely determined by its energy supply, which is difficult to satisfy when the resource requirements of traditional encryption methods are at a high security level [12]. Therefore, the security solutions which demand excessive processing costs are not suitable for WSN [13].

With these challenges, several novel efficient approaches have been explored. Aysal and et al. [14] proposed that sensors can intentionally generate errors in the transmitted data to confuse eavesdroppers. They assume that the statistics of the induced error pattern is only available to the AFC, and the eavesdroppers who do not know this, fail to detect the real state from the eavesdropped data. In [15], the sensor outputs are randomly flipped through XOR operations on the local observations and independent binary variables. It is shown that using carefully selected operation parameters, we can hinder the EFC from making correct decisions while maintaining an acceptable error rate at the AFC. In [16], the sensor outputs are randomly mapped to other possible quantification levels according to a probability matrix. It is assumed that the AFC is aware of the matrix but the EFC is not. Therefore, the AFC can optimize its decision result along with the mapping probabilities while imposing a high error rate on the EFC. In [17], the authors developed a security scheme based on sensor censorship to maximize the ***J***-divergence of the EFC while ensuring that the divergence of the AFC is zero. In [18], the authors proposed a lightweight encryption scheme whereby sensors flip their local decisions based on the instantaneous channel (to the AFC) fading gains which are unknown to the EFC due to the independence of the physical communication channels. They showed that information-theoretic perfect secrecy can be achieved in the condition that the global flipping and non-flipping data have the same size. However, the channel state information (CSI) changes from time to time, if the data flipping of each sensor is independently decided by its local CSI statistic, it will be difficult to satisfy the required equivalent condition. Moreover, currently most of the security schemes are designed for the binary hypothesis testing problems in which only two opposite candidate states are considered. However, in many practically-oriented applications, the target systems may contain multiple states and quantification scales. Thus a more general security model with a high efficiency is required to ensure the data confidentiality of WSN.

In the paper, we proposed a security data propagation method based on pseudo-random encryption (SDPR) for WSN. The underlying idea is that since most of the random functions generate stochastic sequence in a pseudo-random way, such that as long as the input initial random seed is the same, the functions will always return a same random digital sequence; it is nearly impossible to guess the generated sequence without the seed, even when the employed function is known. Furthermore, the time complexity of the pseudo-random function is much lower than traditional high security encryption algorithms, which makes them more applicable to the resource constrained environment of WSN. It should be noted that pseudo-functions are utilized to control the data interfering process in our method SDPR. The detailed discussion of pseudo-functions is out of the scope of the paper, but anyone interested in it can refer to [19,20,21].

Firstly the typical security problem of distributed binary hypothesis testing in WSN is considered. It is assumed that the sensors report their measurements in the form of binary bits and sequentially transmit them to the AFC. At the beginning of a sensing cycle, the AFC and each sensor $s_{i}$ select an unique seed (for pseudo-random functions) based on the channel state information. In each duplex time, $s_{i}$ is randomly assigned to the flipping or non-flipping group based on the output of its random function. The sensors in the non-flipping group directly transmit their binary results to the AFC, while the others perform data encryption in a way to flip their quantized bits (zero turns to one and vice versa). Therefore, the AFC can recover the flipped data while the EFC can not, and the flipping will only prevent the EFC from fusing the correct result. Note that the seeds can be safely decided by the sensors and AFC based on the observation of the main channel state information. In [22,23,24], the wireless channel gains are considered as common randomness exclusively shared by the transmitter and legitimate receiver using the time-division duplexing (TDD) protocol. Specifically, in [18], the magnitude of the channel gain (MCG) is applied to the negotiation of secret keys. As the main channels (sensors to the AFC) and eavesdropping channels (sensors to the EFC) are statistically independent when the EFC is located more than a half wavelength apart from both the sensors and AFC, it is impossible for the EFC to eavesdrop the seed information.

The scheme is then extended to a more common scenario with multiple quantification scales and candidate states. Our goal is to achieve data confidentiality of WSN by ensuring that the data observed by the EFC is useless for detection purposes. It should be noted that the proposed approach has minimal processing requirements and does not introduce any communication overhead.

## 2. The Description of the Security Model

### 2.1. System Model

The proposed security scheme SDPR for binary hypothesis testing is referred to as SDPR_B. The sensors observe a common target state which can be $\theta_{0}$ or $\theta_{1}$, and the measurement of each sensor $s_{i}$ is quantized into binaries (local decision $u_{i}$) based on the measurement and decision threshold: If the measurement is less than the decision threshold, then $u_{i}$ = 1 ($s_{i}$ detected $\theta_{1}$), otherwise $u_{i}$ = –1 ($s_{i}$ detected $\theta_{0}$). Let $p(u_{i}=1|\theta_{1})$ and $p(u_{i}=1|\theta_{0})$ denote the local detection rate $pd_{i}$ and false alarm rate $pf_{i}$ of the *i*-th sensor respectively. Usually, the environment sensing performance $pd_{i}$ and $pf_{i}$ can be known in advance both by the AFC and EFC. In order to prevent the EFC from detecting the real state, the sensors in the flipping group flipped their binary outputs before sending to the AFC.

### 2.2. Flipping Encryption

A sensor is assigned to the flipping group if the output of its local pseudo-random function rand(·) is in the flipping domain $\left[\tau_{4},\tau_{3}\right]$. Usually the output sequence of rand(·) is decided by its initial seed. Therefore, in order to make sure that the AFC and sensors can synchronously generate the same random sequences at the beginning of each sensing cycle, the AFC and each corresponding sensor $s_{i}$ decide $seed_{i}$ based on the observation of the instantaneous channel state of the *i*-th main channel ($s_{i}$ to AFC), such that $seed_{i}$ = $g_{i}$, where $g_{i}$ can be the magnitude of the channel gain or the CQI (channel quality indicator) level. As $g_{i}$ is only known by the AFC and $s_{i}$ due to the channel independence between the main channels and the eavesdropping channels [18], the EFC cannot eavesdrop $seed_{i}$ through the captured data in WSN.

Before the data transmission in each duplex time, the AFC first broadcasts pilot signals with flipping thresholds: $\left\{\tau_{1},\tau_{2},\tau_{3},\tau_{4}\right\}$, where $\left\{\tau_{1}>\tau_{2}>\tau_{3}>\tau_{4}\right\}$, to trigger the sensors to report their local decisions in a time-division duplex (TDD) manner. In particular, the thresholds remain constant during a duplexing time for pilot transmission and sensors’ transmissions, say one block, and changes independently across blocks and sensors. Once the *i*-th sensor received the pilot signal, it immediately generates a random value $\varphi_{i}^{\left(t\right)}$ by rand(·) as shown in Equation (1):

(1) $$\varphi_{i}^{\left(t\right)}=\left\{\begin{array}{cc} rand(seed_{i}), & \;if\;t=1\\ rand(\varphi_{i}^{\left(t-1\right)}), & \;if\;t>1 \end{array}\right.$$

The encrypted data (output) of the *i*-th sensor $s_{i}$ is denoted by $x_{i}$. If $\tau_{2}<\varphi_{i}^{\left(t\right)}<\tau_{1}$, then $s_{i}$ is put into the non-flipping group and output $x_{i}=u_{i}$. On the contrary, $s_{i}$ is assigned to the flipping group when $\tau_{4}<\varphi_{i}^{\left(t\right)}<\tau_{3}$, and its output is $x_{i}=-u_{i}$, which is a bit-flipping version of the quantized data. The remaining sensors are dormant. Note that the EFC can also receive the sensor outputs through eavesdropping channels due to the broadcast nature of WSN. We emphasize that since the encryption is based on the pseudo-random functions’ outputs, which are different between sensors, the EFC cannot distinguish the flipped data from the original outputs even if it has eavesdropped the pilot packets from the AFC. As a result, we expect that the EFC fails to detect the target state. In the next section, we will show that the AFC can correctly fuse the received data whereas the EFC totally failed to utilize the captured data.

### 2.3. The Fusion Result of the AFC

In this section, the fusion result of the AFC based on log-likelihood ratio (LLR) is analyzed. Suppose the number of activated sensors in one duplexing time is k(k≤N), then the input vector, which contains data from all the activated sensors, for the decision fusion is $z^{A}=\left[z_{1}^{A}\ldots z_{k}^{A}\right]$, and according to [25] the LLR-based fusion rule at the AFC is given by:

(2) $$\begin{array}{c} \Lambda =log\frac{P(z^{A}|\theta_{1})}{P(z^{A}|\theta_{0})}=\sum_{i=1}^{k}log\frac{f(z_{i}^{A}|\theta_{1})}{f(z_{i}^{A}|\theta_{0})}\;\begin{array}{c} \theta_{1}\\ ≷\\ \theta_{0} \end{array}\;0 \end{array}$$

f(.|.) is the conditional probability density function. As the sensor outputs have been randomly flipped, $z_{i}^{A}$ may not be the original sensor quantification. Note that the AFC holds the initial $seed_{i}$ of each sensor. Then the original quantized measurement $u_{i}^{A}$ of $z_{i}^{A},i=1\ldots k$, can be recovered by Equation (3):

(3) $$u_{i}^{A}=\left\{\begin{array}{cc} sign(z_{i}^{A}), & if\;\tau_{2}<\varphi_{i}^{\left(t\right)}=rand\left(\varphi_{i}^{\left(t-1\right)}\right)<\tau_{1}\\ -sign(z_{i}^{A}), & if\;\tau_{4}<\varphi_{i}^{\left(t\right)}=rand\left(\varphi_{i}^{\left(t-1\right)}\right)<\tau_{3} \end{array}\right.$$

where sign($z_{i}^{A}$) represents the sign of $z_{i}^{A}$. Let $S_{1}=\left\{i|z_{i}^{A}=1,\tau_{2}<\varphi_{i}^{\left(t\right)}<\tau_{1}\right\}$, $S_{2}=\left\{i|z_{i}^{A}=-1,\tau_{2}<\varphi_{i}^{\left(t\right)}<\tau_{1}\right\}$, $S_{3}=\left\{i|z_{i}^{A}=1,\tau_{4}<\varphi_{i}^{\left(t\right)}<\tau_{3}\right\}$, and $S_{4}=\left\{i|z_{i}^{A}=-1,\tau_{4}<\varphi_{i}^{\left(t\right)}<\tau_{3}\right\}$. Then the left side of Equation (2) can be rewritten as:

(4) $$\begin{array}{c} \Lambda =\sum_{i\in S_{1}}log\frac{f(z_{i}^{A}|\theta_{1})}{f(z_{i}^{A}|\theta_{0})}+\sum_{i\in S_{2}}log\frac{f(z_{i}^{A}|\theta_{1})}{f(z_{i}^{A}|\theta_{0})}+\\ \sum_{i\in S_{3}}log\frac{f(z_{i}^{A}|\theta_{1})}{f(z_{i}^{A}|\theta_{0})}+\sum_{i\in S_{4}}log\frac{f(z_{i}^{A}|\theta_{1})}{f(z_{i}^{A}|\theta_{0})} \end{array}$$

When $i\in S_{1}$, it means that the original quantification $u_{i}^{A}$ is not flipped and $u_{i}^{A}=z_{i}^{A}=1$, therefore the LLR value of the *i*-th sensor in $S_{1}$ becomes:

(5) $$\sum_{i\in S_{1}}log\frac{f(z_{i}^{A}|\theta_{1})}{f(z_{i}^{A}|\theta_{0})}=\sum_{i\in S_{1}}log\frac{f(u_{i}^{A}=1|\theta_{1})}{f(u_{i}^{A}=1|\theta_{0})}=\sum_{i\in S_{1}}log\frac{pd_{i}}{pf_{i}}$$

In the same manner, we can compute the LLR values in different set $S_{2}∼S_{4}$, finally the LLR based fusion rule can be reduced to the following one:

(6) $$\begin{array}{cc} \Lambda & =log\frac{f(z^{A}|\theta_{1})}{f(z^{A}|\theta_{0})}\;\\ & =\sum_{i\in S_{1}}log\frac{pd_{i}}{pf_{i}}+\sum_{i\in S_{2}}log\frac{1-pd_{i}}{1-pf_{i}}+\;\\ & \sum_{i\in S_{3}}log\frac{1-pd_{i}}{1-pf_{i}}+\sum_{i\in S_{4}}log\frac{pd_{i}}{pf_{i}}\;\begin{array}{c} \theta_{1}\\ ≷\\ \theta_{0} \end{array}\;0\; \end{array}$$

Note that this approximation can be viewed as a modified version of the Chair–Varshney fusion rule, which only utilizes local false alarm and detection probabilities [25].

## 3. Security Analysis

In the security analysis of the proposed scheme, the main concern is whether the EFC can get useful information from the eavesdropped data. At the EFC, the captured data from the *i*-th sensor is denoted by $z_{i}^{E}$ and the input vector for EFC’s fusion rule is $z^{E}=\left[z_{1}^{E},z_{2}^{E}\ldots z_{k}^{E}\right]$. And the final decision of EFC is is shown in Equation (7).

(7) $$\begin{array}{c} L=log\frac{f(z^{E}|\theta_{1})}{f(z^{E}|\theta_{0})}\;\begin{array}{c} \theta_{1}\\ ≷\\ \theta_{0} \end{array}\;0 \end{array}$$

From Equation (7) we can see that data confidentiality can be achieved by deriving $f(z^{E}|\theta_{1})$ equals $f(z^{E}|\theta_{0})$, which makes the LLR value at the EFC always equals to zero, and the EFC will totally ignore the data since it cannot make a final decision for the binary hypothesis testing problem when *L* = 0.

Obviously, the sensor outputs are independent with each other, and $f\left(z^{E}|\theta_{1}\right)=\prod_{i=1}^{k}f\left(z_{i}^{E}|\theta_{1}\right)$. Then $f(z_{i}^{E}|\theta_{1})$ is computed with the total probability theorem, as shown in Equation (8).

(8) $$\begin{array}{cc} f\left(z_{i}^{E}|\theta_{1}\right) & =\sum_{u_{i}}\sum_{x_{i}}f\left(z_{i}^{E},x_{i},u_{i}|\theta_{1}\right)\;\\ & =\sum_{u_{i}}p\left(u_{i}|\theta_{1}\right)\sum_{x_{i}}\int f\left(z_{i}^{E},\varphi_{i},x_{i}|u_{i},\theta_{1}\right)d\varphi_{i}\;\\ & =\sum_{u_{i}}p\left(u_{i}|\theta_{1}\right)\sum_{x_{i}}\int f\left(z_{i}^{E}|\varphi_{i},x_{i},u_{i},\theta_{1}\right)\cdot \;\\ & f\left(\varphi_{i}\right)\cdot p\left(x_{i}|\varphi_{i},u_{i},\theta_{1}\right)d\varphi_{i}\; \end{array}$$

where $\varphi_{i}$ denotes the value of the pseudo-random function rand(·) with probability density function $f(\varphi_{i})$, $u_{i}$ and $x_{i}$ represent the original measurement and final output of the *i*-th sensor $s_{i}$ respectively. $z_{i}^{E}$ is conditionally independent of $u_{i}$, $\varphi_{i}$, and $\theta_{1}$ when $x_{i}$ is known, while $x_{i}$ is conditionally independent of $\theta_{1}$ when $\varphi_{i},u_{i}$ are known. Remember that only the sensors whose random values $\varphi_{i}$ belong to $\left[\tau_{2},\tau_{1}\right]$ or $\left[\tau_{4},\tau_{3}\right]$ are activated in a duplex time, and we can derive $f(z_{i}^{E}|\theta_{1})$ as follows:

(9) $$\begin{array}{cc} f(z_{i}^{E}|\theta_{1})= & \sum_{u_{i}}p\left(u_{i}|\theta_{1}\right)\sum_{x_{i}}f\left(z_{i}^{E}|x_{i}\right)\cdot \\ & (\int_{\tau_{2}}^{\tau_{1}}f\left(\varphi_{i}\right)\cdot p\left(x_{i}|\varphi_{i},u_{i}\right)d\varphi_{i}+\\ & \int_{\tau_{4}}^{\tau_{3}}f\left(\varphi_{i}\right)\cdot p\left(x_{i}|\varphi_{i},u_{i}\right)d\varphi_{i}) \end{array}$$

Since the EFC cannot be aware of $\varphi_{i}$ and its probability density function $f(\varphi_{i})$, it should take into account all the flipping cases: (1) $\varphi_{i}\in \left[\tau_{2},\tau_{1}\right],u_{i}=\pm 1,x_{i}=u_{i};$ (2) $\varphi_{i}\in \left[\tau_{4},\tau_{3}\right],u_{i}=\pm 1,x_{i}=-u_{i}$. To simplify the expression, let $\lambda_{1}$ and $\lambda_{2}$ represent $\int_{\tau_{2}}^{\tau_{1}}f\left(\varphi_{i}\right)\cdot p\left(x_{i}|\varphi_{i},u_{i}\right)d\varphi_{i}$ and $\int_{\tau_{4}}^{\tau_{3}}f\left(\varphi_{i}\right)\cdot p\left(x_{i}|\varphi_{i},u_{i}\right)d\varphi_{i}$ respectively, then we have:

(10) $$\begin{array}{cc} f(z_{i}^{E}|\theta_{1}) & =f\left(z_{i}^{E}|x_{i}=-1\right)\left(1-pd_{i}\right)\lambda_{1}\\ & +f\left(z_{i}^{E}|x_{i}=1\right)pd_{i}\lambda_{1}\\ & +f\left(z_{i}^{E}|x_{i}=1\right)\left(1-pd_{i}\right)\lambda_{2}\\ & +f\left(z_{i}^{E}|x_{i}=-1\right)pd_{i}\lambda_{2} \end{array}$$

If $\lambda_{1}$ and $\lambda_{2}$ satisfy $\lambda_{1}=\lambda_{2}=\lambda$, then,

(11) $$f\left(z_{i}^{E}|\theta_{1}\right)=f\left(z_{i}^{E}|x_{i}=1\right)\lambda +f\left(z_{i}^{E}|x_{i}=-1\right)\lambda$$

In the same manner, we can compute $f(z_{i}^{E}|\theta_{0})$ as follows,

(12) $$f\left(z_{i}^{E}|\theta_{0}\right)=f\left(z_{i}^{E}|x_{i}=1\right)\lambda +f\left(z_{i}^{E}|x_{i}=-1\right)\lambda$$

From Equation (9) we can see that the AFC can easily ensure $\lambda_{1}=\lambda_{2}$ as long as the width $\tau_{1}-\tau_{2}=\tau_{3}-\tau_{4}$. And when $\lambda_{1}=\lambda_{2}=\lambda$, it means that the transmitted data from the flipping and non-flipping groups arrive at the EFC with the same size, and the proposed transmission scheme achieves $f\left(z_{i}^{E}|\theta_{1}\right)=f\left(z_{i}^{E}|\theta_{0}\right)$. Consequently, the EFC cannot make the final decision for the nature state $\theta_{1}$ or $\theta_{0}$, and it has to totally ignore the captured data.

## 4. Generalization to the Multiple Decisions

Besides the binary hypothesis testing problem discussed in previous sections, there are many WSN applications involved multiple states $\Theta =\{\theta_{1},\theta_{2}\ldots \theta_{m}\}$, and the sensor quantifications also have multiple scales $\Xi =\{\upsilon_{1},\upsilon_{2}\ldots \upsilon_{n}\}$. Usually each scale corresponds to an unique conditional probability in different state. Then the AFC needs to make a decision from the multiple candidate states while the EFC also tries to eavesdrop the natural state based on the captured data.

Suppose the received vector is $Z=\left[z_{1},z_{2}\ldots z_{k}\right],z_{i}\in \Xi$, according to Bayes rules [26], the state which has the maximum posterior probability is selected as the final decision.

(13) $$\max_{j}\frac{P\left(Z|\theta_{j}\right)P\left(\theta_{j}\right)}{\sum_{i=1}^{n}P\left(Z|\theta_{i}\right)P\left(\theta_{i}\right)}$$

Without loss of generality, we assume that the prior probability of each state is equal, therefore Equation (13) can be simplified to:

(14) $$\max_{j}\Pi_{i=1}^{k}P\left(z_{i}|\theta_{j}\right)$$

**Figure 1:**
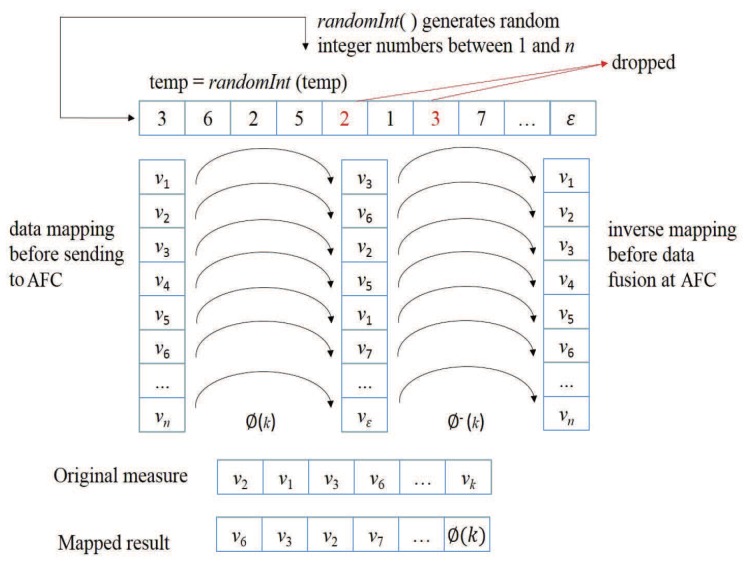
Procedure of pseudo-random encryption (SDPR_M).

In order to prevent the EFC from forming the correct fusion result of Equation (14), the proposed SDPR is extended for the case of multiple scales (referred to SDPR_M). As shown in Figure 1, the measurement results are automatically mapped to other quantification scales based on a 1×n mapping matrix ϕ before sending to the AFC. Consequently, the AFC recovers the received data using an inverse mapping before data fusion. To make sure that the AFC and each sensor $s_{i}$ keep the same mapping matrix $\phi_{i}$, firstly, they select an initial $seed_{i}$ in a similar manner as described in Section 2 for a pseudo-random function *randInt* (usually, $randInt\left(seed_{i}\right)=\left\lfloor min+rand\left(seed_{i}\right)*\left(max-min\right)\right\rfloor$), which uniformly generates random integer numbers in a given range [*min*, *max*] based on the $seed_{i}$, and randInt always outputs the same sequence of numbers with the same $seed_{i}$. And then, in the *t*-th time-division sensor $s_{i}$ generates a random sequence and forms $\phi_{i}$ using the following process:

- Step 1 initialize $\phi_{i}=null$, R=null, *j* = 2, *n* = number of scales;
- Step 2 $R\left(1\right)=randInt\left(seed_{i}\right)$, temp=R(1);
- Step 3 if j>n update; $seed_{i}=randInt\left(R\left(n\right)\right)$, go to step 5, else temp=randInt(temp), endif;
- Step 4 if ∀k<j,R(k)≠temp,R(j)=temp,j=j+1, endif, go to Step 3;
- Step 5 set $\phi_{i}=\left\{\Xi \left(R\left(1\right)\right),\Xi \left(R\left(2\right)\right)\ldots \Xi \left(R\left(n\right)\right)\right\}$.

As shown in Equation (15), if the quantified result of sensor $s_{i}$ is $u_{i}=\upsilon_{j}$, then the final output $x_{i}$ is mapped to another scale $x_{i}=\phi_{i}\left(j\right)$ based on the mapping matrix.

(15) $$x_{i}=\phi_{i}\left(j\right),\;if\;u_{i}=\upsilon_{j},where\;\phi_{i}\left(j\right),\;\upsilon_{i}\in \Xi$$

After the data mapping, the output is sent to the AFC to form a decision using Equation (14). Then we analyze the security of the mapping schemes for multiple states and scales.

Firstly, it is assumed that the outputs are transmitted through error-free channels, and the received data from the *i*-th sensor is $z_{i}=x_{i}$. Let us consider the probability of $P(z_{i}|\theta_{j})$ in Equation (14).

(16) $$\begin{array}{cc} P(z_{i}|\theta_{j}) & =\sum_{u_{i}}P\left(z_{i},u_{i}|\theta_{j}\right)\\ & =\sum_{u_{i}}P\left(u_{i}|\theta_{j}\right)P\left(z_{i}|u_{i},\theta_{j}\right)\\ & \overset{\left(a\right)}{=}\sum_{u_{i}}P\left(u_{i}|\theta_{j}\right)P\left(\Phi \left(u_{i}\right)=z_{i}|u_{i},\theta_{j}\right)\\ & \overset{\left(b\right)}{=}\sum_{u_{i}}P\left(u_{i}|\theta_{j}\right)P\left(\Phi \left(u_{i}\right)=z_{i}\right) \end{array}$$

where Φ(.) is a mapping function defined on matrix $\phi_{i}$, such that $\Phi \left(\nu_{k}\right)=\nu_{t}$ if $\phi_{i}\left(k\right)=\nu_{t}$ and $P(\Phi \left(u_{i}\right)=z_{i})$ represents the probability of mapping scale $u_{i}$ to $z_{i}$. (a) Follows from the fact that the final output of $s_{i}$ is decided by the local measurement $u_{i}$ and the mapping matrix; (b) follows the fact that the mapping probability of $P(\Phi \left(u_{i}\right)=z_{i})$ is independent of $u_{i}$ and $\theta_{j}$ when the mapping matrix Φ is known. Let $P\left(\Phi \left(u_{i}\right)=z_{i}\right)=\lambda_{i}$, then $P\left(z_{i}|\theta_{j}\right)=\sum_{u_{i}}P\left(u_{i}|\theta_{j}\right)\lambda_{i}$.

As the random function randInt generate integers uniformly between 1 and *n*, it can be assumed that the probability of mapping $\upsilon_{i}$ to any $\upsilon_{j},\;i,j=1,2\ldots n$ is the same and $\lambda_{1}=\lambda_{2}\ldots =\lambda_{n}=\lambda$. Therefore, Equation (16) is simplified as:

(17) $$\begin{array}{cc} P(z_{i}|\theta_{j}) & =\sum_{u_{i}}P\left(u_{i}|\theta_{j}\right)\lambda \\ & =\lambda \end{array}$$

Then we discuss a more complex scenario that the channel is interfered by noise, and for the *i*-th sensor, the received data is given by $z_{i}=h_{i}\cdot x_{i}+n_{i}$, where $h_{i}$ is the channel gain and $n_{i}$ is the signal noise. If the channel is not error-free, $z_{i}$ may not equal $x_{i}$, and $f(z_{i}|\theta_{j})$ in Equation (14) can be derived as follows:

(18) $$\begin{array}{cc} P(z_{i}|\theta_{j}) & =\sum_{u_{i}}\sum_{x_{i}}P\left(z_{i},x_{i},u_{i}|\theta_{j}\right)\\ & =\sum_{u_{i}}P\left(u_{i}|\theta_{j}\right)\sum_{x_{i}}P\left(x_{i}|u_{i},\theta_{j}\right)P\left(z_{i}|x_{i},u_{i},\theta_{j}\right)\\ & \overset{\left(a\right)}{=}\sum_{u_{i}}P\left(u_{i}|\theta_{j}\right)\sum_{x_{i}}P\left(x_{i}|u_{i}\right)P\left(z_{i}|x_{i}\right)\\ & \overset{\left(b\right)}{=}\sum_{u_{i}}P\left(u_{i}|\theta_{j}\right)\sum_{x_{i}}P\left(\Phi \left(u_{i}\right)=x_{i}\right)P\left(z_{i}|x_{i}\right) \end{array}$$

where (a) follows the fact that $z_{i}$ is independent of $u_{i}$ and $\theta_{j}$ when $x_{i}$ is known, and (b) follows from the fact that the output of $x_{i}$ is decided by $u_{i}$ and local mapping matrix. As we have discussed in Equation (17), the probability of mapping $u_{i}$ to any quantification scale can be the same, and $P(z_{i}|\theta_{j})$ is further derived as:

(19) $$\begin{array}{cc} P(z_{i}|\theta_{j}) & =\sum_{u_{i}}P\left(u_{i}|\theta_{j}\right)\sum_{x_{i}}\lambda P\left(z_{i}|x_{i}\right) \end{array}$$

The probability of $P(z_{i}|x_{i})$ in Equation (19) is decided by the physical state of the wireless channel, including channel gains, noise level, etc. However, the physical channel can be assumed to be stable during one duplex time, and $\sum_{x_{i}}P\left(z_{i}|x_{i}\right)$ is a constant value $P_{i}$, which is not related to the candidate state $\theta_{j}$. Finally we can have:

(20) $$\begin{array}{cc} P(z_{i}|\theta_{j}) & =\sum_{u_{i}}P\left(u_{i}|\theta_{j}\right)\sum_{x_{i}}\lambda P\left(z_{i}|x_{i}\right)\\ & =\lambda \cdot P_{i} \end{array}$$

The AFC can recover the original quantifications based on reverse mapping of $\phi_{i}$, but the EFC dose not know the mapping matrix, and has to fuse the raw data using Equation (14). According to Equations (17) and (19), the EFC for any input vector $Z=[z_{1},z_{2}\ldots z_{k}]$ and candidate state $\theta_{j},j=1,2\ldots m$, 1) in error-free channels $P\left(Z|\theta_{j}\right)=\Pi_{i=1}^{k}P\left(z_{i}|\theta_{j}\right)=\lambda^{k}$; 2) in noise channels $P\left(Z|\theta_{j}\right)=\Pi_{i=1}^{k}P\left(z_{i}|\theta_{j}\right)=\lambda^{k}\cdot \Pi_{t=1}^{k}P_{t}$. For both of the two cases, the conditional probability of the fusion rules in Equation (14) is all the same no matter what the candidate state $\theta_{j}$ is. Therefore, it will be nearly impossible for the EFC to generate correct decisions based on the captured sensor data.

## 5. Simulation Results

### 5.1. Experiments on Binary Hypothesis Testing

In this section, a group of simulations were carried out to test whether SDPR_B could prevent the EFC from eavesdropping the real state, while the AFC could correctly fuse the sensor outputs when there were only two candidate states.

The comparison objects include security fusion rules proposed in [18] (referred to Jeon), in which the sensors’ binary decisions are flipped based on instantaneous channel gains in the main channels to the AFC. In addition, the performance of the Optimum-LLR proposed in [26], where the optimum LLR based fusion rule is derived without the presence of the EFC, is considered as the lower bounds of the error probability.

For ease of comparisons, we adopted a similar condition as in [18], the sensors were deployed into a star-like topology and the main channel gains were assumed to follow a Rayleigh distribution. Furthermore, the sensors have the same local detection performances $p_{f}=0.2,\;p_{d}=0.9$. The total error probability by $P_{\varepsilon }=\Delta \left(1-P_{d}\right)+\left(1-\Delta \right)P_{f}$ is taken as the criterion of fusion performance, where $P_{d}$ and $P_{f}$ are the detection and false alarm probabilities at the fusion center, respectively, and Δ=0.5 is a weighting factor.

**Figure 2:**
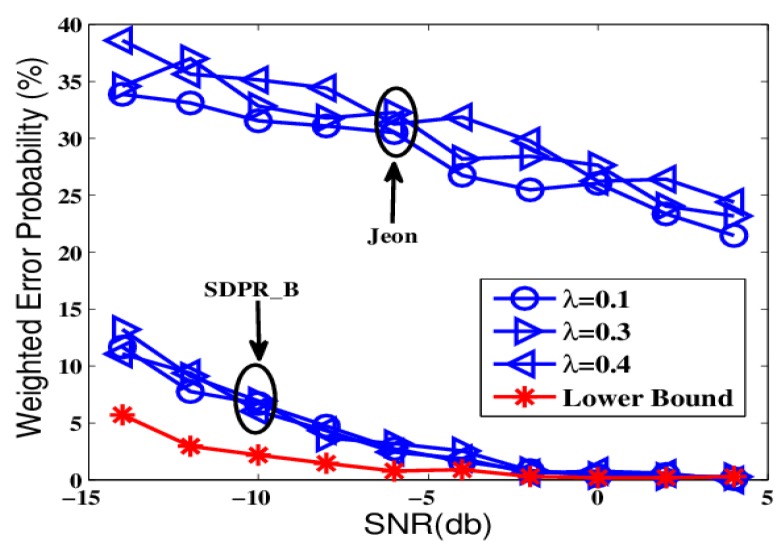
The error probabilities at the ally fusion center (AFC) as a function of signal to noise rate (SNR) in ideal channel conditions.

**Figure 3:**
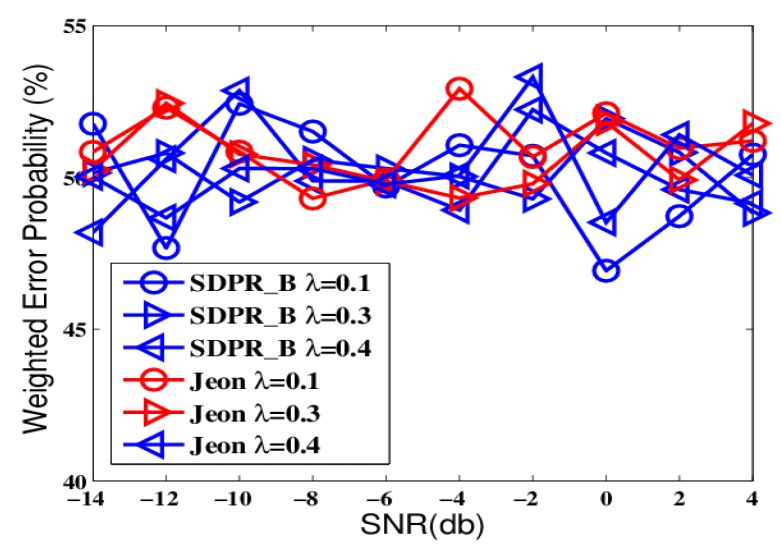
The error probabilities at the enemy fusion center (EFC) as a function of SNR in ideal channel conditions.

**Figure 4:**
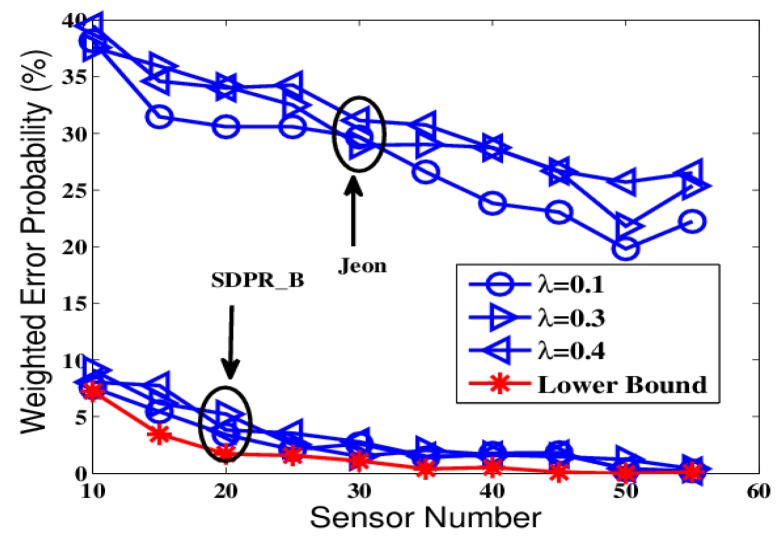
The error probabilities at the AFC with an increasing number of sensors in ideal channel conditions.

The first round of comparisons were carried out in ideal conditions that the channel gains strictly followed the Rayleigh distribution. The results are shown in Figure 2, Figure 3 and Figure 4, which depict the weighted error probability of the AFC and EFC with different signal to noise rate (SNR) and sensor number.

**Figure 5:**
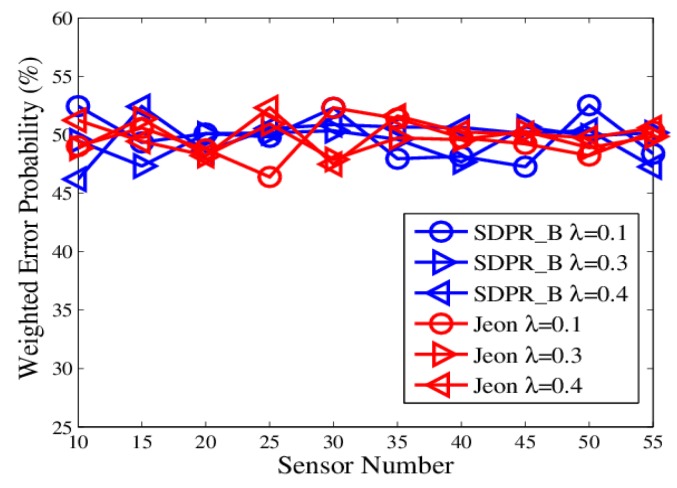
The error probabilities at the EFC with an increasing number of sensors in ideal channel conditions.

From Figure 2 and Figure 4, we can see that Optimum-LLR achieved the lowest error probability (lower bound). However, Optimum-LLR does not take any security mechanism against eavesdropping, thus the EFC can get the same low error rate as that achieved by the AFC. For SDPR_B, its error probability at the AFC was near to that of Optimum-LLR, and obviously better than Jeon. That is because the AFC could recover the flipped data using inverse flipping before data fusion. On the other hand, from Figure 3 and Figure 5, we can see that the error probability of the EFC was always near 50% even with high SNR and many sensors, because it could not distinguish the flipped data from the original outputs, which completely interfered its data fusion. Therefore, in the ideal condition, both SDPR_B and Jeon achieved information-theoretic perfect secrecy.

**Figure 6:**
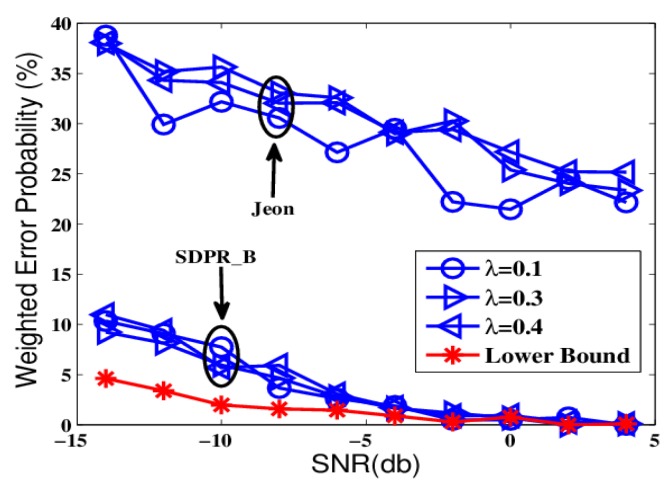
The error probabilities at the AFC as a function of SNR in time-varying channel conditions.

**Figure 7:**
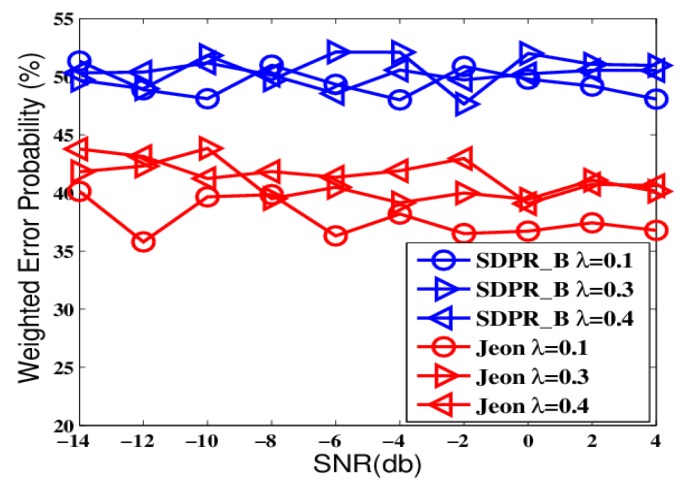
The error probabilities at the EFC as a function of SNR in time-varying channel conditions.

**Figure 8:**
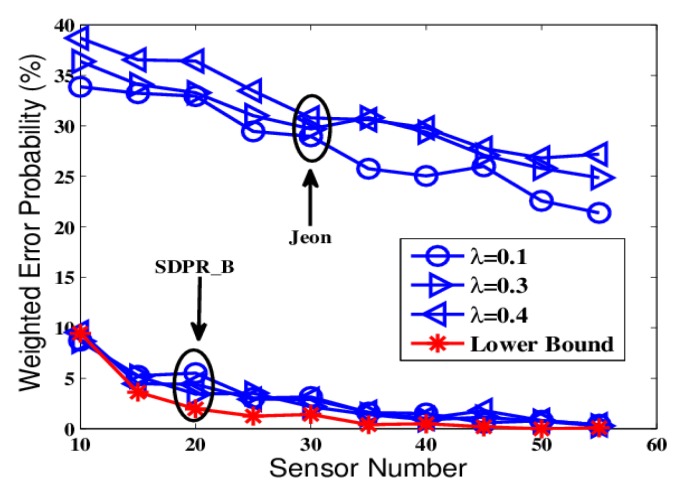
The error probabilities at the AFC with an increasing number of sensors in time-varying channel conditions.

**Figure 9:**
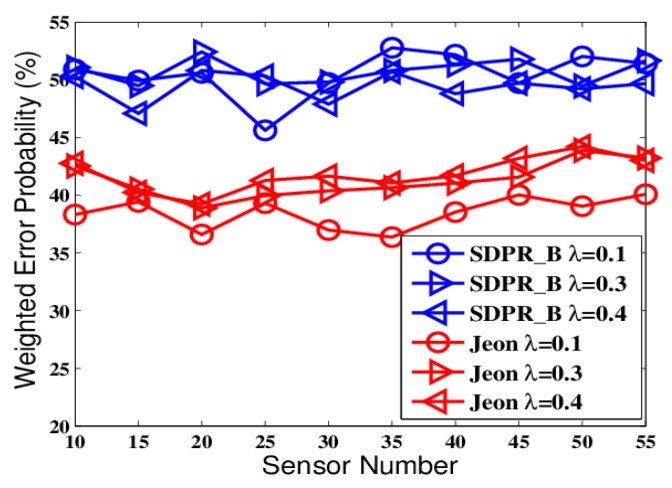
The error probabilities at the EFC with an increasing number of sensors in time-varying channel conditions.

To further test the proposed schemes, we designed another group of comparisons that simulated a more realistic network environment where the channel gains continuously changed and we had no prior-knowledge of the probability distribution of the gains. The results are shown in Figure 6, Figure 7, Figure 8 and Figure 9.

The figures depict that, at the AFC, the error probabilities of SDPR_B and Jeon decrease as the SNR and sensor number increased. Furthermore, for the EFC, the error probability of SDPR_B is always near to 50%, but compared with the results in the ideal condition, the error probability of Jeon apparently decreased. In the real situations, the channel gain, which affects the data flipping of Jeon, dynamically change due to power consumption, external disturbance, etc. Therefore, the numbers of flipping data and non-flipping data of Jeon are not equal any more, resulting in less disturbance to the EFC. As shown in Figure 7 and Figure 9, the average error probability is reduced to 40%, which means the confidentiality of Jeon is compromised. In addition, the data flipping of SDPR_B is controlled by the pre-deployed random function, and when the AFC sets $\tau_{1}-\tau_{2}=\tau_{3}-\tau_{4}$ in Equation (9), it ensures that the sizes of the flipped and non-flipped data in the EFC are the same and the two sets of data are self-contradictory, resulting in the error probability of the EFC to be always near 50%, which is a necessary condition of ideal confidentiality for binary hypothesis testing in WSN.

### 5.2. Experiment on Multiple Scales

In this section, we tested the security schemes involved in multiple scales and states. Our goal is to demonstrate that SDPR_M proposed in the paper can ensure that the fusion result of EFC provides no more information than the priori-knowledge of the natural state, while the AFC can make a correct decision.

**Figure 10:**
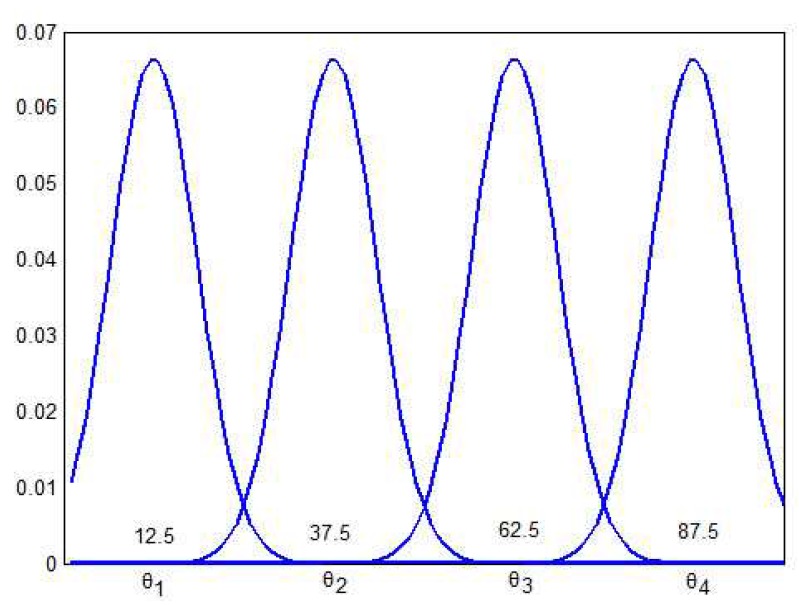
The distribution of sensor quantification in different states.

Our experiment simulated a common scenario in the industry area: *n* sensors quantize the vibration velocity of a mechanical system and periodically report the quantifications to the fusion center through WSN. The velocity scale ranges from S={1,2…100}, while the candidate states consists of $\theta_{1},\theta_{2},\theta_{3},\theta_{4}$, corresponding to ‘Idle’, ‘Busy’, ‘High Load’, and ‘Broken’ respectively. As shown in Figure 10, the sensor quantifications in different state are assumed to follow Gauss distributions, such that $p\left(x|\theta_{1}\right)∼N\left(12.5,8\right)$, $p\left(x|\theta_{2}\right)∼N\left(37.5,8\right)$, $p\left(x|\theta_{3}\right)∼N\left(62.5,8\right)$, $p\left(x|\theta_{4}\right)∼N\left(87.5,8\right)$ and, $p\left(x|\theta_{i}\right)=\int_{x-1}^{x}f{\left(x\right)}_{\theta_{i}}dx$. Without loss of generality, the priori-probability of each state is assumed to be the same.

**Figure 11:**
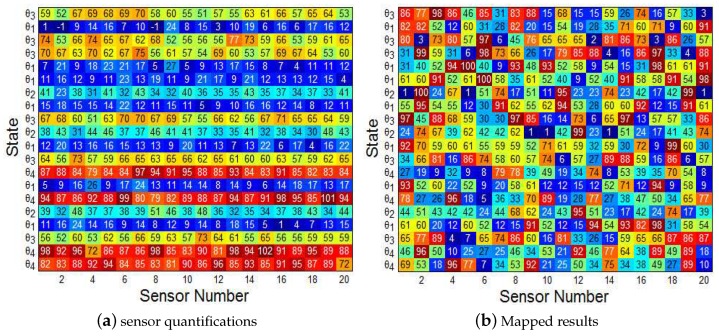
An example of data mapping with 20 sensors and four candidate states *θ*_1_ to *θ*_4_.

In each round of the simulation, one state $\theta_{i},i\in \left\{1,2,3,4\right\}$ is randomly selected and then the sensor measurements $\left[u_{1},u_{2}\ldots u_{n}\right],\;u_{i}\in S$ are generated based on the probability density function $p(x|\theta_{i})$. As described in Section 4, before data transmission, sensor $s_{i},i=1\ldots n$ mapped its measurement $u_{i}$ to another scale based on its mapping matrix to confuse the EFC. An example of data mapping is shown in Figure 11, each rows in (a) represent the original measurements of 20 sensors for a given state $\theta_{i}$, while the rows in (b) are the corresponding mapped results which are totally different from the original ones. Note that the probability of mapping a scale to any one in *S* is the same. The theoretical number of possible mapping matrix is 100!=9.3326215443944e+157 and it is nearly impossible for the EFC to recover the original quantifications through brute-force analyzing of the captured data (it will cost more than 3e+132 years, even if America’s newest top supercomputer ’SUMMIT’ is employed to the analysis).

For comparisons, we employed a native Bayes decision fusion rules [27] which is derived by using the principle of maximum posterior probability without data mapping.

**Figure 12:**
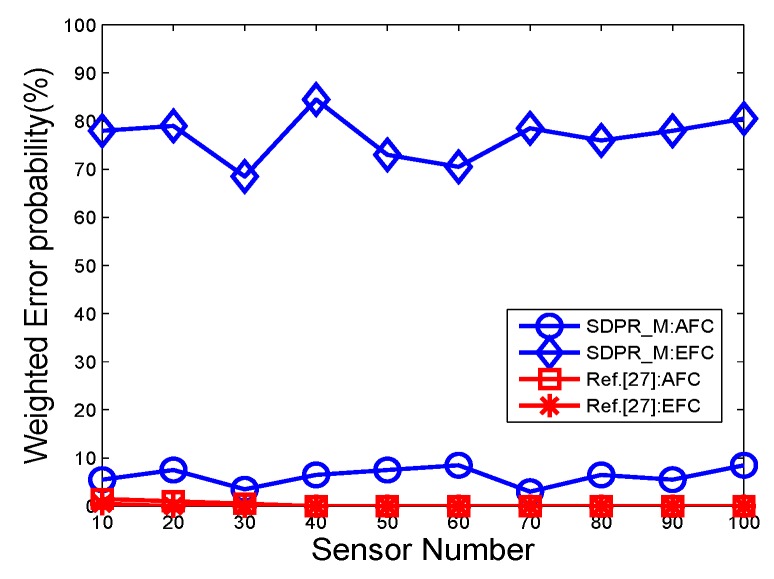
The error probabilities as a function of the number of deployed sensors for SNR = −5 dB.

In the first group of comparisons, 30% of the sensor outputs are interfered by White Gaussian noise. Figure 12 depicts the average error probability with different sensor number for 10 rounds of simulations. We see that in [27] as a lower error bound of the AFC, however the EFC has a similar performance too due to the lack of data encryption. Therefore, the EFC can easily eavesdrop the target state based on the captured data. The figure shows that the AFC of SDPR_M has a similar performance with that of [27]. Meanwhile, the EFC of SDPR_M has an apparently higher error probability and the corresponding true detection rate is near 25%, which is the priori-knowledge of the distribution of the 4 states. That is because in SDPR_M, the AFC and sensors deployed same pseudo-random functions and initial seeds, which ensures that they also have same mapping matrices and the AFC can recover the original quantifications through inverse mapping of the received data. In addition, the initial seeds are selected based on the instantaneous state information of the main channels (sensors to AFC). The EFC is unaware of the mapping matrix because of the independence between the main and eavesdropper channels. Therefore the data mapping only causes interference to the EFC’s fusion results.

**Figure 13:**
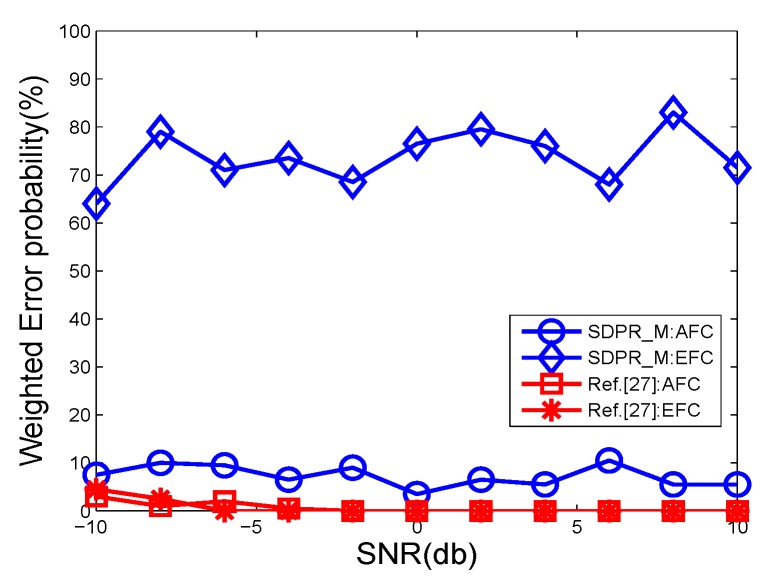
The error probabilities as a function of various SNRs for *N* = 20.

We can see similar results in Figure 13, which depicts the error probability with different SNRs. The results present a similar trend that the AFC of SDPR_M has a performance close to [27], while the EFC can still not get useful information about the state even with high SNR. The experiment confirmed the capabilities of SDPR_M in that it ensures the security of WSN’s open transmissions while reserving the high performance of the AFC.

To see the performance superiority of the proposed scheme, another group of simulations were carried out to compare SDPR_M with the scheme proposed in [16] where the sensor outputs are randomly mapped to other scales using an optimized stochastic cipher matrix Φ. Its encryption is converted to solve the following optimization problem of Φ:

$$\begin{array}{l} \Phi^{*}=\underset{\Phi }{arg}max\;J_{A}(\alpha_{1}\Phi \Delta ||\alpha_{2}\Phi \Delta )\\ \begin{array}{ll} \mathrm{subject}\;\mathrm{to}: & 1)0\leq \phi_{ij}^{*}\leq 1,2)\Phi^{*}1_{m\times 1}=1_{m\times 1},\mathrm{and}\\ & 3)(q_{1}\alpha_{1}+q_{2}\alpha_{2})\Phi^{*}\Delta =1/m1_{1\times m} \end{array} \end{array}$$

where $J_{A}$ is the detection gain of the AFC, $q_{1}$ and $q_{2}$ represent priori-probability of two candidate states $\theta_{i}$, *i* = 1,2, $\alpha_{i}=\left[p\left(x_{1}|\theta_{i}\right),p\left(x_{2}|\theta_{i}\right)\ldots p\left(x_{m}|\theta_{i}\right)\right]$ represents the post-probability vector of multiple scales in state $\theta_{i}$, $\phi_{ij}$ is the probability of mapping a scale *i* to another level *j*, and Δ is the transition probability matrix. In addition, the native Bayes fusion rule [27] without data encryption is also compared and its results are taken as a lower bound of the error probability.

The proposed scheme in [16] is limited to two candidate states. For fair comparisons, it is assumed that there are only two states $\theta_{1}$, $\theta_{2}$, and the quantification scales {1,2…100} are assumed to follow Gauss distributions in different state: $p\left(x|\theta_{1}\right)∼N\left(37.5,12\right)$, and $p\left(x|\theta_{2}\right)∼N\left(62.5,12\right)$.

**Figure 14:**
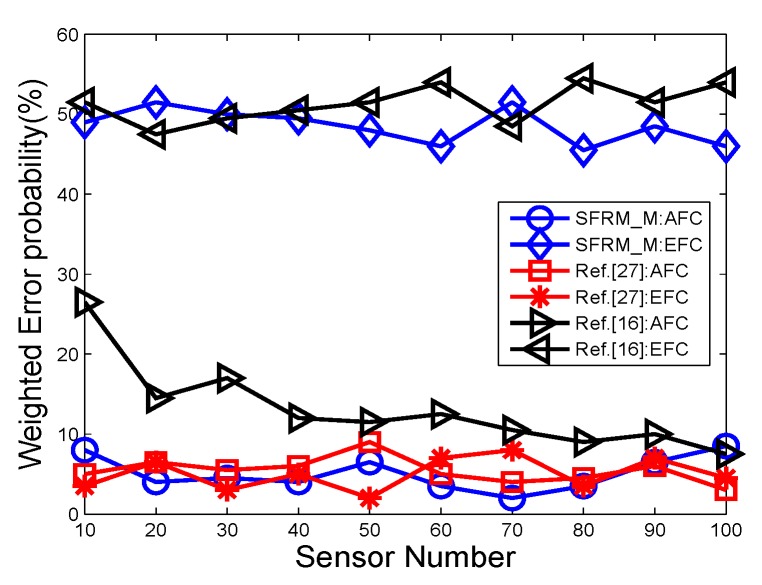
The error probabilities as a function of the number of deployed sensors for SNR = −5 dB.

**Figure 15:**
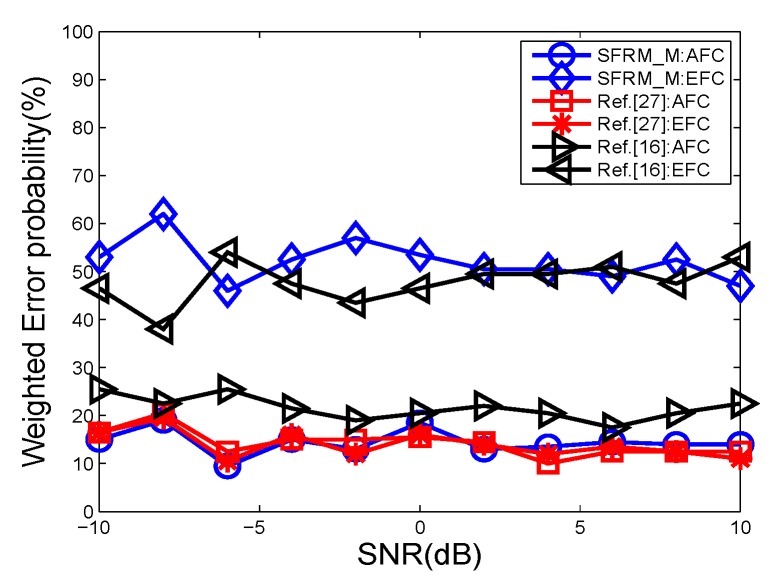
The error probabilities as a function of various SNRs for *N* = 20.

The comparison results are depicted in Figure 14 and Figure 15. From the figures, we can see that both SDPR_M and the scheme proposed in [16] ensure that the error probability is around 50% at the EFC. Furthermore, the performance of SDPR_M at the AFC is quite similar to the lower bound shown as the red line. However, the performance at the AFC of [16] obviously deteriorated, especially when the sensor number is less than 20, the error probability is higher than 20%. That is because the optimization problem of the stochastic cipher matrix Φ in [16] is NP-hard, and usually its heuristical solutions are suboptimal, which results in the non-trivial degeneration of performance across a wide range of sensor numbers (SNR values). Although the scheme in [16] achieved good confidentiality, it sacrificed the performance of the AFC. Whereas SDPR_M realized a similar security level that maintained data availability of the AFC. In addition, it should be noted that in many applications, both the data confidentiality and the data reliability should be taken into consideration. Therefore, from this point of view our method can be more applicable.

## 6. Conclusions

In the paper, a lightweight scheme was proposed to protect data confidentiality in a distributed sensor network. The data was supposed to be transmitted over open and insecure channels with the presence of an enemy fusion center EFC, which tried to gather all the transmitted data to form its own decision regarding the state of nature. To prevent the EFC from eavesdropping, the security scheme was designed by exploiting randomness of data flipping (mapping). The main idea was that the activated sensors change their quantized outputs in a random way based on pseudo-random functions known by sensors and the AFC. Thus the EFC who captured the sensor data over the open channels failed to perform data decryption since it could not distinguish the original output from the flipped data. The theory analysis and experimental results demonstrated that the AFC could appropriately decrypt the data, but for the EFC, even with high SNR and large number of sensors, it still could not make right decisions on the state.

We claim that due to the simplicity and low complexity, the proposed solution could be deployed in many resource-limited applications of WSN, including natural disaster monitoring, battlefield situation awareness, remote control of unmanned aerial vehicle, etc. Furthermore, the new pseudo-encryption model opens several future research lines. Being a generalization of data-flipping encryption, we can expect stochastic data flipping to allow better confidentiality while reserving data utility. Exploring the capability limit of pseudo random flipping for data confidentiality is another possible follow-up of this article. Finally, it should be noted that our method is preferred in the condition that the number of sensor quantification scales were stable and known in advance, and how to improve the proposed method for the unpredictable and unstable environment of WSN will be taken into consideration in our future work.

## References

[1] Reyes-Menendez A., Palos-Sanchez P., Saura J.R., Martin-Velicia F. (2018). Understanding the Influence of Wireless Communications and Wi-Fi Access on Customer Loyalty: A Behavioral Model System. Wirel. Commun. Mob. Com..

[2] Saura J.R., Reyes-Menendez A., Palos-Sanchez P. (2019). Mapping multispectral Digital Images using a Cloud Computing software: Applications from UAV images. Heliyon.

[3] Shigen S., Hongjie L., Risheng H., Athanasios V.V., Yihan W., Qiying C. (2014). Differential game-based strategies for preventing malware propagation in wireless sensor networks. IEEE Trans. Inf. Forensics Secur..

[4] Murat D., Yunus O., Cevat B. (2015). Fire detection systems in wireless sensor networks. Procedia Soc. Behav. Sci..

[5] Huang D.J., Teng W.C. (2015). A defense against clock skew replication attacks in wireless sensor networksoriginal research article. J. Netw. Comput. Appl..

[6] He D., Chan S., Guizani M. (2015). Accountable and privacy–enhanced access control in wireless sensor networks. IEEE Trans. Inf. Forensics Secur..

[7] Soltanmohammadi E., Oroojiand M., Naraghi–Pour M. (2013). Decentralized hypothesis testing in wireless sensor networks in the presence of misbehaving nodes. IEEE Trans. Inf. Forensics Secur..

[8] Moosavi H., Bui F.M. (2014). A game–theoretic framework for robust optimal intrusion detection in wireless sensor networks. IEEE Trans. Inf. Forensics Secur..

[9] Uluagac A.S., Beyah R.A., Copeland J.A. (2013). Secure source–based loose synchronization (sobas) for wireless sensor networks. IEEE Trans. Parallel Distrib. Syst..

[10] Yanrong L., Lixiang L., Haipeng P., Yixian Y. (2016). An Energy Efficient Mutual Authentication and Key Agreement Scheme Preserving Anonymity for Wireless Sensor Networks. Sensors.

[11] Dawei Z., Haipeng P., Lixiang L., Yixian Y. (2014). A secure and effective anonymous authentication scheme for roaming service in global mobility networks. Wirel. Pers. Commun..

[12] Akyildiz I.F., Su Y.S.W., Cayirci E. (2002). Wireless sensor networks: A survey. Comput. Netw..

[13] Incebacak D., Bicakci K., Tavli B. (2015). Evaluating energy cost of route diversity for security in wireless sensor networksoriginal. Comput. Stand. Interfaces.

[14] Aysal T.C., Barner K. (2008). Sensor data cryptography in wireless sensor networks. IEEE Trans. Inf. Forensics Secur..

[15] Pour M.N., Nadendla V. Secure detection in wireless sensor networks using a simple encryption method. Proceedings of the IEEE Wireless Communications and Networking Conference (WCNC).

[16] Soosahabi R., Naraghi–Pour M., Perkins D., Bayoumi M.A. (2014). Optimal probabilistic encryption for secure detection in wireless sensor networks. IEEE Trans. Inf. Forensics Secur..

[17] Marano S., Matta V., Willett P.K. (2009). Distributed detection with censoring sensors under physical layer secrecy. IEEE Trans. Signal Process..

[18] Jeon H., Choit J., McLaughlin S.W., Ha J. (2013). Channel aware encryption and decision fusion for wireless sensor networks. IEEE Trans. Inf. Forensics Secur..

[19] Matsumoto M., Nishimura T. (1998). Mersenne Twister: A 623–Dimensionally Equidistributed Uniform Pseudo–Random Number Generator. ACM Trans. Model. Comput. Simul..

[20] Naor M. (2004). Number–Theoretic Constructions of Efficient Pseudo–Random Functions. J. ACM.

[21] Wichmann B.A., Hill I.D. (1982). An Efficient and Portable Pseudo–random Number Generator. J. R. Stat. Soc. Ser. C Appl. Stat..

[22] Liu Y., Draper S.C., Sayeed A.M. (2012). Exploiting channel diversity in secret key generation from multipath fading randomness. IEEE Trans. Inf. Forensics Secur..

[23] Wallace J., Sharma R. (2010). Automatic secret keys from reciprocal MIMO wireless channels: Measurement and analysis. IEEE Trans. Inf. Forensics Secur..

[24] Ye C., Mathur S., Reznik A., Shah Y., Trappe W., Mandayam N. (2010). Information–theoretically secret key generation for fading wireless channels. IEEE Trans. Inf. Forensics Secur..

[25] Varshney P. (2005). Distributed Detection and Data Fusion.

[26] Chen B., Jiang R., Kasetkasem T., Varshney P.K. (2006). Channel aware decision fusion in wireless sensor networks. IEEE Trans. Signal Process..

[27] Guerriero M., Svensson L., Willett P. (2010). Bayesian Data Fusion for Distributed Target Detection in Sensor Networks. IEEE Trans. Signal Process..

